# High dietary selenium intake is associated with less insulin resistance in the Newfoundland population

**DOI:** 10.1371/journal.pone.0174149

**Published:** 2017-04-05

**Authors:** Yongbo Wang, Meiju Lin, Xiang Gao, Pardis Pedram, Jianling Du, Chandurkar Vikram, Wayne Gulliver, Hongwei Zhang, Guang Sun

**Affiliations:** 1 Department of Endocrinology, the First Affiliated Hospital of Dalian Medical University, Dalian, Liaoning, China; 2 Department of Biliary Minimally Invasive Surgery, the Affiliated Zhongshan Hospital of Dalian University, Dalian, Liaoning, China; 3 Discipline of Medicine, Faculty of Medicine, Memorial University of Newfoundland, St. John’s, Newfoundland, Canada; 4 Division of Endocrinology, Faculty of Medicine, Memorial University of Newfoundland, St. John’s, Newfoundland, Canada; University of Hawai'i at Manoa College of Tropical Agriculture and Human Resources, UNITED STATES

## Abstract

As an essential nutrient, Selenium (Se) is involved in many metabolic activities including mimicking insulin function. Data on Se in various biological samples and insulin resistance are contradictory, moreover there is no large study available regarding the relationship of dietary Se intake with insulin resistance in the general population. To investigate the association between dietary Se intake and variation of insulin resistance in a large population based study, a total of 2420 subjects without diabetes from the CODING (Complex Diseases in the Newfoundland Population: Environment and Genetics) study were assessed. Dietary Se intake was evaluated from the Willett Food Frequency questionnaire. Fasting blood samples were used for the measurement of glucose and insulin. Insulin resistance was determined with the homeostasis model assessment (HOMA-IR). Body composition was measured using dual energy X-ray absorptiometry. Analysis of covariance showed that high HOMA-IR groups in both males and females had the lowest dietary Se intake (μg/kg/day) (p < 0.01), being 18% and 11% lower than low HOMA-IR groups respectively. Insulin resistance decreased with the increase of dietary Se intake in females but not in males after controlling for age, total calorie intake, physical activity level, serum calcium, serum magnesium, and body fat percentage (p < 0.01). Partial correlation analysis showed that dietary Se intake was negatively correlated with HOMA-IR after adjusting for the Se confounding factors in subjects whose dietary Se intake was below 1.6 μg/kg/day (r = -0.121 for males and -0.153 for females, p < 0.05). However, the negative correlation was no longer significant when dietary Se intake was above 1.6 μg/kg/day. Our findings suggest that higher dietary Se intake is beneficially correlated with lower insulin resistance when total dietary Se intake was below 1.6 μg/kg/day. Above this cutoff, this beneficial effect disappears.

## Introduction

Selenium (Se) is an essential micronutrient element, and a key component of several selenoproteins with essential enzymatic functions that include redox homeostasis [1], thyroid hormone metabolism [2], protection from oxidative stress [3] and inflammation [1,3,4]. Data from a good number of studies suggested Se was associated with the development of type 2 diabetes (T2DM) [5,6], but the findings are contradictory.

As early as 1990s, data from isolated rat adipocytes suggest that Se (as selenate) can mimic the effects of insulin, including stimulating glucose transport activity and enhancing insulin receptor kinase activity [7]. Since then, a number of cell and animal studies have provided evidence that Se has an important role in regulating glucose homeostasis [8–12]. Based on these findings and the potential of selenoproteins to protect against oxidative stress [13], the expectation that Se might be protective against T2DM arose. However, results from human observational studies using a variety of biological samples were inconsistent. Some studies identified positive association between levels of Se in serum/plasma/dietary/fingernails and increased risk of T2DM [14–19], conversely, others found negative association between toenail Se and prevalence of T2DM [20,21], or no significant association [22,23]. Moreover, results from randomized clinical Se supplementation trials have raised concern that high Se exposure might increase the development of T2DM [24–27], but the results were still contradictory [6].

The association between Se nutritional status and diabetes is very complicated and intriguing. Insulin resistance is not only a hallmark but also a pathogenic factor of T2DM. However, the quantitative relationship between dietary Se intake and insulin resistance has been only reported in studies with very small sample size [28,29] or special groups such as metabolic syndrome (MS) [30], obesity [31,32] and polycystic ovary syndrome (PCOS) patients [33]. A study with large sample size and a wide range of insulin resistance and dietary Se intake in a general population is required in order to understand the association between the two factors. More importantly, many major confounding factors that could potentially affect the results were poorly controlled in these reported studies. Although dietary Se intake is the major source of Se for the body, Se nutritional status was overwhelmingly evaluated using hair or serum Se rather than dietary Se intake in reported studies. Consequently, the evidence linking dietary Se intake and insulin resistance is lacking.

Therefore, we designed the present study to investigate the association between dietary Se intake and insulin resistance in a large general population with systematic control of major confounding factors.

## Subjects and methods

### Subjects

**Fig 1:**
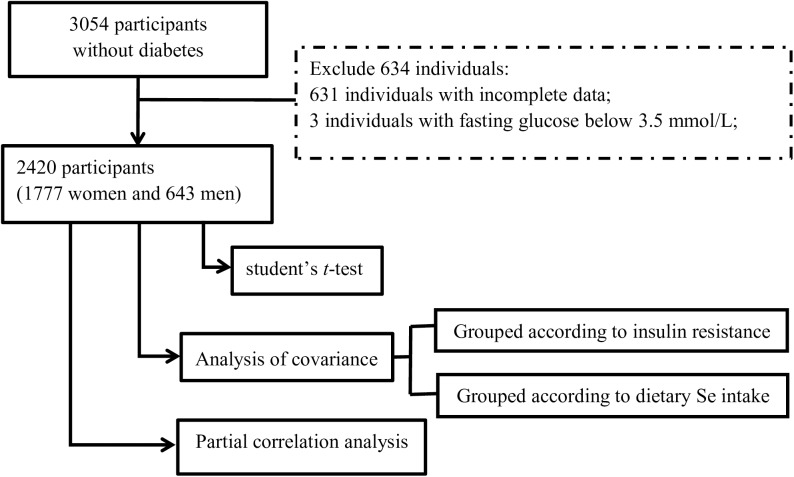
Flow-chart of subjects selection for analyse.

All participants were from the ongoing CODING (Complex Diseases in the Newfoundland Population: Environment and Genetics) study. Eligibility of participants for the CODING study was based upon the following inclusion criteria: 1) ≥19 years of age; 2) at least a third generation Newfoundlander; 3) without serious metabolic, cardiovascular or endocrine diseases; 4) women were not pregnant at the time of the study [34–38]. Ethics approval was obtained from the Health Research Ethics Authority (HREA), Memorial University, St. John’s, Newfoundland, Canada, with Project Identification Code #10.33 (latest date of approval: February 14, 2016). All subjects provided written and informed consent before participation in this study. Detailed information regarding the CODING Study was reported in our previously published papers [34–38]. Subjects with diabetes were excluded to focus on the non-diabetic general population and to avoid the effect of diabetic state on the results. Diabetes mellitus (DM) was defined as fasting blood glucose (FBG) ≥ 7.0 mmol/L or clinically diagnosed DM. A total of 3054 participants without diabetes were initially identified. Among them 631 individuals with incomplete data were excluded, and another 3 were excluded because their fasting glucose were lower than 3.5 mmol/L that resulted in negative values for Homeostatic Model Assessment of β-cell function (HOMA-β). Therefore, 2420 individuals (1777 females and 643 males) were included in the present study (Fig 1).

### Anthropometric measurements

Anthropometrics were performed following a 12-hour overnight fast. Trained personnel obtained these measurements for each subject using standard procedures. Standing height was measured using a fixed stadiometer (to the nearest 0.1 cm). After fully emptying their bladders, subjects wore standard hospital gowns for all weight measurements using a platform manual scale balance (Health O Meter, Bridgeview, IL; nearest 0.1 kg). Body mass index (BMI) (kg/m^2^) was calculated as weight in kilograms divided by height squared in meters. Waist circumference (WC) was defined as the midway point between the iliac crest and the lowest rib, and hip by the maximum circumference over the buttocks below the iliac crest. Waist-hip ratio (WHR) was the division of WC by hip circumference.

### Body composition measurements

Body compositions including total body fat percentage (BF%), trunk fat percentage (TF%), android fat percentage (AF%) and gynoid fat percentage (GF%) were measured, in a supine position, utilizing dual energy X-ray absorptiometry (DXA: Lunar Prodigy; GE Medical Systems, Madison, WI) with the Lunar Prodigy software system. The Lunar Prodigy software system has the capacity to distinguish each of these regions. Trunk fat region is from the top of the shoulders to the top of the iliac crest, while the android fat region is the top of the second lumbar vertebra to the top of the iliac crest and the gynoid fat region extends down the iliac crest twice the height of the android area. The enCORE (Ver 12.2, 2008, GE Medical Systems, Madison, WI) software package was used for DXA data acquisition. Daily quality assurance was performed on the DXA scanner and the typical coefficient of variation was 1.3% during the study period [34–38].

### Dietary assessment

Dietary intake of each participant was assessed using a 124 item semi- quantitative Willett food frequency questionnaire (FFQ) [39,40]. The Willett FFQ obtains from subjects, the number of weekly servings consumed of common food items over the past 12 months. The NutriBase Clinical Nutrition Manager (version 8.2.0; Cybersoft Inc, Phoenix, AZ) software package was used to convert weekly serving values into mean daily serving values. This information was then used to calculate the total daily calorie, macro- and micro-nutrients intakes including Se for each individual [34]. Then dietary Se intake (μg/kg/day) was calculated by dividing body weight [34].

### Physical activity assessment and other information

All participants completed a self-administered screening questionnaire, which was used to collect information of personal health history. Physical activity patterns were measured using the ARIC Baecke Questionnaire, which consists of a Work Index, Sports Index, and Leisure Time Activity Index [34, 41].

### Biochemical measurements

Venous blood samples were collected in the morning after an overnight fast (12 hours). Serum samples were isolated from whole blood and stored at −80°C for subsequent analysis. FBG were measured on an Lx20 analyzer (Beckman Coulter Inc., Fullerton, CA) using Synchron reagents. Fasting insulin (FINS) was measured on an Immulite Immunoassay analyzer. Insulin resistance and β cell function were determined with the homeostasis model assessment (HOMA-IR and HOMA-β), as described by Matthews et al [42].

HOMA-IR = (Fasting Insulin [mU/L]×Fasting Glucose [mmol/L])/22.5

HOMA-β = (20×Fasting Insulin [mU/L])/(Fasting Glucose [mmol/L]—3.5)

### Data analyses

All data are presented as mean ± standard error of the mean(SEM). FINS, HOMA-IR, HOMA-β, calorie intake and dietary Se intake were log-transformed in order to normalize data distributions to perform effective statistical analysis. Anthropometrics, body composition, dietary intake and biochemical measurements were compared between females and males with independent Student's t-test.

In order to analyze the variation of dietary Se intake in different status of insulin resistance, participants were divided into tertiles (low, medium, and high) of insulin resistance based upon HOMA-IR. Dietary Se intake was compared among the three groups with analyses of variance and covariates (ANCOVA) controlling for age, calorie intake and physical activity. The variations of insulin resistance in different dietary Se intakes were analyzed after participants were divided into tertiles (low, medium, and high dietary Se intake). FBG, FINS, HOMA-IR, and HOMA-β were compared among groups using ANCOVA controlling for age, calorie intake, physical activity, serum calcium, serum magnesium, and BF%. Serum calcium and magnesium were taken into consideration as well because our previous studies have shown that they were associated with insulin resistance [35, 43].

Dietary Se intake below 0.4 μg/kg/day is considered as Se deficiency [44]. The relationships between dietary Se intake and HOMA-IR, HOMA-β were further analyzed, after subjects were divided into 10 groups by an interval of 0.4 μg/kg/day (≤0.4, 0.4–0.8, 0.8–1.2, 1.2–1.6, 1.6–2.0, 2.0–2.4, 2.4–2.8, 2.8–3.2, 3.2–3.6, 3.6–4.0 μg/kg/day). The number of subjects with dietary Se intake > 4.0 μg/kg/day was too small (n = 38, female/male = 27/11) to perform an effective statistical analysis. Therefore, they were excluded from the analysis.

Partial correlation analysis, controlling for age, calorie intake, physical activity, serum calcium, serum magnesium, and BF%, was subsequently applied to further confirm the findings from ANCOVA. To control for possible influence of smoking, alcohol drinking, disease status medication use and menopausal, analyses were performed in participants with and without these confounding factors.

All statistical analyses were performed using SPSS 20.0 (SPSS Inc., Chicago, IL). All tests were two sided and p < 0.05 was considered to be statistically significant.

## Results

### Clinical and dietary Se characteristics of the study subjects

Clinical and dietary characteristics of the study subjects are presented in Table 1. Female subjects were on average 3.6 years older than male subjects. Weight, BMI, WC, WHR, FBG, FINS, HOMA-IR, physical activity, serum calcium, and serum magnesium in males were significantly greater and HOMA-β, TF%, AF%, GF%, BF% were significantly lower than females (p < 0.01 for all). In terms of dietary intake, male participants had a significantly higher calorie and Se intake (μg/day) than female participants (p < 0.01), because of the large body size and weight in males. However, after body weight was adjusted (μg/kg/day), there was no significant difference between males and females (p = 0.19).

**Table 1:** Clinical characteristics and dietary Se intake according to gender.

	Entire cohort (n = 2420)	Females (n = 1777)	Males (n = 643)	p value
Age (yr)	42.40 ± 0.27	43.42 ± 0.27	39.80 ± 0.47	< 0.001
Weight (kg)	74.04 ± 0.34	69.16 ± 0.29	86.64 ± 0.53	< 0.001
BMI (kg/m^2^)	26.56 ± 0.10	26.14 ± 0.11	27.67 ± 0.16	< 0.001
WC (cm)	91.57 ± 0.29	89.35 ± 0.29	97.32 ± 0.45	< 0.001
WHR	0.91 ± 0.001	0.89 ± 0.001	0.97 ± 0.002	< 0.001
TF%	36.11 ± 0.19	38.43 ± 0.19	30.11 ± 0.33	< 0.001
AF%	41.15 ± 0.23	43.20 ± 0.23	35.87 ± 0.40	< 0.001
GF%	40.08 ± 0.20	44.51 ± 0.14	28.59 ± 0.27	< 0.001
BF%	33.82 ± 0.19	37.18 ± 0.17	25.14 ± 0.28	< 0.001
FBG (mmol/L)	5.02 ± 0.01	4.96 ± 0.01	5.17 ± 0.02	< 0.001
FINS (pmol/L)	67.61 ± 0.89	65.77 ± 0.99	72.6 1± 1.88	0.008
HOMA-IR	2.24 ± 0.03	2.17 ± 0.04	2.43 ± 0.07	< 0.001
HOMA-β	133.84 ± 1.98	136.54 ± 2.56	126.39 ± 3.72	0.002
Serum calcium (mmol/L)	2.36 ± 0.002	2.35 ± 0.002	2.38 ± 0.003	< 0.001
Serum magnesium (mmol/L)	0.88 ± 0.002	0.88 ± 0.001	0.89 ± 0.002	< 0.001
Physical activity	8.28 ± 0.03	8.18 ± 0.03	8.53 ± 0.06	< 0.001
calorie intake (kcal/day)	1991.55 ± 18.49	1873.63 ± 17.62	2317.87 ± 38.32	< 0.001
Se (μg/day)	109.22 ± 1.18	102.34 ± 1.11	128.23 ± 2.78	< 0.001
Se (μg/kg/day)	1.53 ± 0.02	1.53 ± 0.02	1.53 ± 0.03	0.19

All data presented as mean ± SEM. BMI, Body mass index; WC, Waist circumference; WHR, Waist hip rate; TF%, trunk fat percentage; AF%, android fat percentage; GF%, gynoid fat percentage; BF%, total body fat percentage; FBG, fasting blood glucose; FINS, fasting insulin; HOMA-IR, homeostasis model assessment of insulin resistance; HOMA-β, homeostasis model assessment of β cell function.

### Comparison of dietary Se intake among groups with low, medium and high insulin resistance

Significant differences of dietary Se intake were revealed between the three groups with different insulin resistance after controlling for age, total calorie intake and physical activity (Table 2). Compared with low HOMA-IR group, dietary Se intakes in medium and high HOMA-IR groups were 6%, 11% lower in males, and 8%, 18% lower correspondingly in females. The significant differences were confirmed in pairwise comparisons (p < 0.05), except between male low and medium HOMA-IR group (p = 0.24).

**Table 2:** Comparison of Se intake based on the levels of insulin resistance.

		Low	Medium	High	p trend
Females		592	592	592	-
	HOMA-IR	0.40~1.42	1.42~2.32	2.32~24.07	-
	Se (μg/day)	102.19 ± 1.34	99.84 ± 1.32	101.23 ± 1.35	0.21
	Se (μg/kg/day)	1.62 ± 0.02	1.49 ± 0.02	1.34 ±0.02	< 0.001
Males		214	214	214	
	HOMA-IR	0.40~1.60	1.60~2.74	2.74~16.37	-
	Se (μg/day)	117.70 ± 3.15	117.83 ± 3.02	121.83 ± 3.10	0.62
	Se (μg/kg/day)	1.47 ± 0.04	1.38 ± 0.04	1.30 ±0.04	< 0.001

Data were assessed with Covariance controlling for age, total calorie intake, and physical activity. All values are presented as means ± SEMs. HOMA-IR, homeostasis model assessment of insulin resistance.

### Comparison of insulin resistance in subjects with different dietary Se intake groups

When subjects were grouped into tertiles according to dietary Se intake (low, medium and high), levels of FINS, HOMA-IR, HOMA-β presented a dose-dependent decline (high, medium and low) with the increase of dietary Se intake after controlling for age, total calorie intake, physical activity, serum calcium, serum magnesium and BF% in females (p < 0.05) not in males (Table 3).

**Table 3:** Insulin resistance according to dietary Se intake.

		Low	Medium	High	p
Female	Number	592	592	592	-
	Se (μg/kg/day)	0.16 ~1.12	1.22 ~ 1.66	1.66 ~ 8.89	-
	FBG (mmol/L)	5.05 ± 0.02	4.99 ± 0.02	4.99 ± 0.02	0.13
	FINS (pmol/L)	73.02 ± 1.86	62.47 ± 1.59	61.23 ± 1.96	<0.001
	HOMA-IR	2.41 ± 0.07	2.02 ± 0.06	2.04 ± 0.08	<0.001
	HOMA-β	142.22 ± 4.36	131.40 ± 3.72	126.77 ± 4.59	0.01
Male	Number	214	214	214	-
	Se (μg/kg/day)	0.22 ~ 1.05	1.05 ~ 1.61	1.61 ~ 7.19	-
	FBG (mmol/L)	5.25 ± 0.04	5.23 ± 0.03	5.27 ± 0.04	0.69
	FINS (pmol/L)	78.24 ± 3.88	69.76 ± 3.34	73.61 ± 4.62	0.30
	HOMA-IR	2.63 ± 0.14	2.36 ± 0.12	2.51 ± 0.17	0.28
	HOMA-β	129.75 ± 8.57	122.21 ± 7.37	135.42 ± 10.19	0.41

Data were assessed with covariates controlling for age, calorie intake, physical activity, serum calcium, serum magnesium, and BF%. Data presented as mean ± SEM. FBG, fasting blood glucose; FINS, fasting insulin; HOMA-IR, homeostasis model assessment of insulin resistance; HOMA-β, homeostasis model assessment of β cell function.

For each 1 μg/kg/day increase in dietary Se intake, average weight, BMI, WC, and WHR decreased by 8.39 kg, 2.98 kg/m^2^, 8.03 cm, and 0.02 in women, and by 8.85 kg, 2.34 kg/m^2^, 7.39 cm, and 0.02 in men, respectively. Likewise, TF%, AF%, GF% and BF% were reduced by 4.58%, 5.56%, 3.05% and 4.16% in women, and by 5.43%, 5.94%, 4.19% and 4.45% in men, respectively (Table 3). Dietary Se intake (μg/kg/day) alone accounted for 9–27% of the variations in body fat.

**Fig 2:**
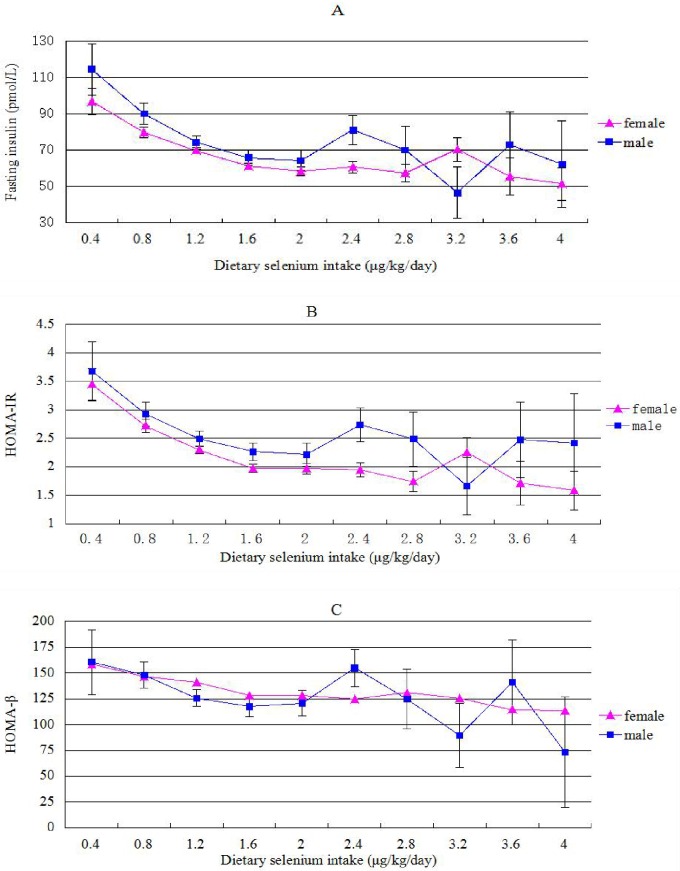
Variations in fasting insulin, HOMA-IR and HOMA-β with increasing dietary Se intake. HOMA-IR, homeostasis model assessment of insulin resistance; HOMA-β, homeostasis model assessment of β cell function.

Subjects were divided into 10 groups based on dietary Se intake with an interval of 0.4 μg/kg/day, and the variations of FINS, HOMA-IR and HOMA-β with dietary Se intake were analyzed with covariates (Fig 2). As dietary Se intake increased from 0.4 to 1.6 μg/kg/day, FINS, HOMA-IR, HOMA-β decreased correspondingly in a pattern close to linear relationship for both genders. However this linear pattern came to a plateau, and disappeared from the level of 1.6 to 4.0 μg/kg/day in both genders.

### Correlations between dietary Se intake and insulin resistance by sex

The correlations between dietary Se intake and FINS, HOMA-IR and HOMA-β are presented in Table 4. When dietary Se intake was ≤ 1.6 μg/kg/day, in both male and female subjects, dietary Se intake (μg/kg/day) was significantly negatively correlated with FINS, HOMA-IR and HOMA-β (r0 was from -0.107 to -0.227, p < 0.001). The correlations remained significant (r1 was from -0.102 to -0.153, p < 0.05) after adjusting for age, total calorie intake, physical activity, serum calcium, serum magnesium and BF% in both genders. When dietary Se intake was greater than 1.6 μg/kg/day, the negative correlations disappeared after controlling these confounding factors in both genders.

**Table 4:** Correlation of dietary Se intake with insulin resistance.

Dietary Se intake (μg/kg/day)		≤ 1.6		> 1.6	
		r_0_ (p)	r_1_(p)	r_0_ (p)	r_1_(p)
Females	FBG	-0.157 (0.000)	-0.103 (0.001)	-0.042 (0.303)	-0.048 (0.240)
	FINS	-0.183 (0.000)	-0.148 (0.000)	-0.028 (0.492)	-0.049 (0.238)
	HOMA-IR	-0.191 (0.000)	-0.153 (0.000)	-0.036 (0.375)	-0.064 (0.121)
	HOMA-β	-0.107 (0.000)	-0.102 (0.001)	-0.001 (0.981)	-0.003 (0.939)
Males	FBG	-0.165 (0.001)	0.022 (0.664)	-0.112 (0.150)	-0.043 (0.586)
	FINS	-0.216 (0.000)	-0.124 (0.014)	-0.177 (0.023)	-0.004 (0.958)
	HOMA-IR	-0.227 (0.000)	-0.121 (0.017)	-0.186 (0.016)	-0.015 (0.853)
	HOMA-β	-0.156 (0.002)	-0.121 (0.017)	-0.111 (0.154)	0.041 (0.606)

Partial correlations between dietary Se intake (μg/kg/day) and insulin resistance were controlling for age, total caloric intake, physical activity, serum calcium, serum magnesium, and body fat percentage. FBG, fasting blood glucose; FINS, fasting insulin; HOMA-IR, homeostasis model assessment of insulin resistance; HOMA-β, homeostasis model assessment of β cell function. r0: correlation coefficient; r1: partial correlation coefficient.

To further exclude the influence of additional covariates, data of participants who were non-smokers, non-drinkers, on no medication and otherwise healthy were analyzed. In Table 5, for each subgroup, partial correlation analyses were conducted controlling for age, total calorie intake, physical activity, serum calcium, serum magnesium and BF%. Dietary Se intake remained negatively correlated with HOMA-IR.

**Table 5:** Partial correlations between dietary Se intake and body compositions excluding the effect of Smoking, drinking, medication use and disease.

	No Smoking		No Alcohol		No Medication		No Disease	
	r1	r2	r1	r2	r1	r2	r1	r2
Female								
FBG	-0.086**	-0.045	-0.078	0.055	-0.081*	0.012	-0.128**	-0.065
FINS	-0.137**	-0.038	-0.179**	-0.062	-0.106*	-0.012	-0.133**	-0.042
HOMA-IR	-0.138**	-0.055	-0.180**	-0.078	-0.110*	-0.015	-0.140**	-0.061
HOMA-β	-0.101**	0.010	-0.139*	-0.027	-0.075*	-0.014	-0.082*	-0.007
Male								
FBG	-0.063	-0.065	-0.154	0.268	-0.027	-0.035	-0.010	-0.046
FINS	-0.137*	-0.024	-0.279*	0.220	0.105*	-0.061	-0.104*	-0.014
HOMA-IR	-0.136*	-0.037	-0.280*	0.211	-0.096*	-0.048	-0.098*	-0.024
HOMA-β	-0.122*	0.031	-0.238	0.233	0.124	-0.095	-0.112	0.030

Partial correlations between dietary Se intake (μg/kg/day) and insulin resistance controlling for age, total caloric intake, physical activity, serum calcium, serum magnesium, and body fat percentage. FBG, fasting blood glucose; FINS, fasting insulin; HOMA-IR, homeostasis model assessment of insulin resistance; HOMA-β, homeostasis model assessment of β cell function. r1: partial correlation coefficient, when dietary Se intake was below or equal to 1.6 μg/kg/day; r2: partial correlation coefficient, when dietary Se intake was greater than 1.6 μg/kg/day.

*, P<0.05

** P<0.01.

## Discussion

In the present study, we analyzed the associations between dietary Se intake and insulin resistance in the large CODING study with a wide range of dietary Se intake among 2,420 adult Newfoundlanders. To the best of our knowledge, this is the first large cross-sectional study specifically designed to analyze the association between dietary Se intake and insulin resistance in the general population. The most important finding is that dietary Se intake was significantly negatively associated with insulin resistance in females and males after controlling all major confounding factors, when dietary Se intake was ≤ 1.6 μg/kg/day most subjects fell in this range. However, when dietary Se intake was > 1.6 μg/kg/day, this reverse relationship was no longer significant. The findings suggest that the beneficial negative relationship between dietary Se intake and insulin resistance may have a dose ceiling.

Se may affect insulin resistance via multiple routes including insulin-like action, inflammatory cytokines and oxidative stress. Early studies showed that sodium selenate may mimic insulin to stimulate glucose uptake [7–9]. In addition, Se may reduce insulin resistance through inhibiting the activity and the production of inflammatory cytokines including tumour necrosis factor-a (TNF-a), nuclear factor-kappa B (NF-κB) and interleukin (IL-1 and IL-18) [45–49]. On the other hand, roles of reactive oxygen species (ROS) in insulin signaling depend on the balance of ROS production and antioxidant defense [50]. Both excessive ROS [51–53] and excessive consumption of ROS [32, 33] are involved in insulin resistance. Adequate Se intake as a potent antioxidant can decrease ROS and improve insulin resistance. However higher Se intake can increase the expression of selenoproteins including GPx, which may induce insulin resistance by removing hydrogen peroxide [54–57]. The ceiling phenomenon of dietary Se intake discovered from the present study may provide further evidence demonstrating the changing effect on insulin resistance with increase the of dietary Se intake.

Insulin resistance is a complex pathophysiological condition. There are numerous factors that can potentially be involved in the development of insulin resistance. It is critical to identify and properly control or adjust major factors in a large population study because if these factors are not properly adjusted, they would potentially cause either false positive or negative results. Similarly a variety of factors may potentially affect dietary Se intake. Food choice and consumption and insulin resistance vary among different age and gender groups [58–61], making age and gender primarily important confounding factors to be adjusted. Dietary calorie intake as a general indicator of nutrient intake is positively correlated with dietary Se intake [34] and negatively with insulin sensitivity [62]. Because physical activity [63], body fat accumulation [64], Serum magnesium [35] and calcium [43] are also associated with insulin resistance, they were adjusted as potential confounding factors in our analyses. Smoking, alcohol drinking, concomitant illness and medication use (including Se supplement) are all potentially important covariates of insulin resistance. Even after these covariates were excluded, the association between dietary Se intake and insulin resistance remained significant. One of major strengths in the present study is the systematic control of these confounding factors, ensuring the findings in the present study are more accurate and reliable.

Previous studies had reported conflicting results for the relationships between Se in various biological samples and insulin resistance. It was reported that hair Se were negatively correlated with the HOMA-IR controlled for age and sex in a small Korean study [28]. A weak negative correlation between serum Se level and HOMA-IR was found in first-degree relatives of diabetic patients in Turkey [29]. However, in a Swedish study including 1024 elderly men, serum Se level was not associated with insulin resistance after adjusting for age, BMI, cigarette smoking, leisure time physical activity and education [23]. Contrarily positive correlations between serum Se level and HOMA-IR were reported in elderly Polish men with MS [30] and obese Egyptian children [31]. Administration of 200 μg/day Se supplements for 6 weeks resulted in a significant decrease in serum insulin and HOMA-IR levels among women with central obesity [32] and PCOS [33]. However, there is no large study available focusing on dietary Se intake and insulin resistance in the general population. The present study filled the knowledge gap, and revealed that dietary Se intake is indeed associated with low insulin resistance suggesting a beneficial effect of dietary Se on insulin sensitivity.

Another important finding from the present study is the ‘threshold’ of the beneficial effect of dietary Se intake on insulin resistance. The beneficial association generally started from very low level to 1.6 μg/kg/day, approximately equivalent to 118 μg/day for person with average body weight (139 μg/day for males and 110 μg/day for females). This beneficial association was nearly linear within this range but became weaker and disappeared if dietary Se intake was above this level. This cut-off point is similar to the suggested dietary Se dose (100–150 μg/day) for tumor protection [65]. This cut-off point will be essential for determining the most appropriate dose of Se supplementation required to maximize its beneficial effect and to avoid potential adverse effects. Existing data suggest that both Se deficiency and over supplementation may be associated with insulin resistance [65, 66] and increased the risk of type 2 diabetes [6].Previous studies have reported a trend of U-shaped association between serum Se and risk of cardiovascular diseases [67], serum Se and serum triglycerides [68]. The association between dietary Se and insulin resistance revealed in the present study seems to be U-shaped as well. Se supplementation will be beneficial to insulin resistance for those with a Se level below 1.6 μg/kg/day. However, Se supplement may be unnecessary for most people without significant low dietary Se intake. Caution has to be applied in Se supplementation as excessive amount potentially have harmful effects. It must be noted that the cutoff point used in the present study was obtained from statistical analysis in the Newfoundland population. More studies in different populations are warranted.

There are several potential limitations in the present study. Although the study has identified and adjusted many major confounding factors, it is inevitably other potential factors were not included, such as zinc and copper which have been reported to have effects on insulin sensitivity [69, 70], however, the data of zinc and copper was not available at the time when analyses were performed. In addition, dietary Se cannot completely represent the Se nutrition status [71]. Other potential metrics of Se nutrition status such as serum Se level and selenoproteins will be measured and analyzed in the near future. In addition, dietary Se exists in two forms: inorganic (selenate and selenite) and organic (selenomethionine and selenocysteine). The nature of the dietary questionnaires did not allow us to evaluate further and identify Se chemical form that exist in the foods, which needs to measure in special method. It is hard to evaluate/separate the data also by predominant selenium chemical forms that exist in the foods in a large sample study. Hence, the effect of different Se chemical forms on insulin resistance cannot be distinguished either. Finally, a cross-sectional design does not allow the determination if dietary Se intake is a causal effect for reduced insulin resistance. Direct intervention study using Se supplement among patients with insulin resistance and low level of dietary Se will be warranted.

## Conclusions

In conclusion, our findings revealed a significantly negative association of dietary Se intake with insulin resistance in the large CODING study with many major confounding factors adjusted, when dietary Se intake was below 1.6 μg/kg/day. Given the narrow margin between Se deficiency, adequacy, over-nutrition and toxicity, accurately determining this cutoff point is clinically important. People with an adequate or high Se status have already received the maximum benefit from Se and need not take additional Se supplementation, because of the potential risks, such as the increased risk of type 2 diabetes. Ultimately, this study aims to add to our present knowledge about the optimal constitution of an ideal diet to reduce insulin resistance and risk of type 2 diabetes in terms of specific macronutrient and micronutrient composition.
